# Mechanisms of Zooplankton Community Assembly and Their Associations with Environmental Drivers in Arid-Region Reservoirs of Northwest China

**DOI:** 10.3390/biology14060732

**Published:** 2025-06-19

**Authors:** Xuelian Qiu, Fangze Zi, Long Yun, Qiang Huo, Liting Yang, Yong Song, Shengao Chen

**Affiliations:** 1 College of Life Sciences and Technology, Tarim Research Center of Rare Fishes, State Key Laboratory Incubation Base for Conservation and Utilization of Bio-Resource in Tarim Basin, Tarim University, Alar 843300, China; 10757231124@stumail.taru.edu.cn (X.Q.); 10757213076@stumail.taru.edu.cn (F.Z.); 10757232147@stumail.taru.edu.cn (L.Y.); 10757223082@stumail.taru.edu.cn (Q.H.); 10757231127@stumail.taru.edu.cn (L.Y.); 2 College of Material Science and Engineering, Beijing University of Chemical Technology, Beijing 100029, China

**Keywords:** arid regions, zooplankton functional groups, driving factors, assembly mechanisms

## Abstract

This study investigates the mechanisms of zooplankton community assembly and their relationship to environmental factors in high-latitude arid regions. We conducted seasonal sampling at four reservoirs in the upper Tarim River Basin from 2023 to 2024: Shangyou Reservoir (SY), Shengli Reservoir (SL), Duolang Reservoir (DL) and Xinjingzi Reservoir (XJZ). The zooplankton community was categorized into five functional groups based on the predominant species, with small crustacean filter feeders (SCF) in all reservoirs except XJZ, where a seasonal shift between rotifer collectors (RC) in the wet season and SCF in the dry season was observed. Pearson correlation and canonical correspondence analysis (CCA) revealed that interspecific competition, pH, conductivity (COND), and salinity (SALIN) were the main determinants of zooplankton community composition. Significant correlations (*p* < 0.05) were detected among functional groups RC (rotifers carnivora), RF (rotifers filter feeders), SCF (small copepods and claocera filter feeders), and MCC (middle copepods and claocera carnivora). Environmental factors showed significant spatial heterogeneity, while zooplankton biomass was positively correlated with pH and COND. Cluster similarity analyses indicated complex interactions between 29 zooplankton species, with RF identified as an important positive predictor for larger groups. The network of co-occurrences showed predominantly positive relationships, emphasizing the mutual facilitation between the species. Our results suggest that interspecific interactions have stronger effects on community structuring than environmental factors, with mutual facilitation emerging as an important survival strategy. This study provides important insights into the dynamics of zooplankton communities in dry reservoirs and establishes a framework for understanding ecological patterns and assembly mechanisms under drought conditions.

## Simple Summary

This study examined the community structure and its relationship with environmental factors in four representative reservoirs located in the Tarim River Basin, Xinjiang, China. A total of 85 zooplankton species were identified, with rotifers exhibiting the highest species diversity, while cladocerans demonstrated the largest density and biomass. The zooplankton were categorized into five functional groups, with the SCF group being predominant. The study found that pH and salinity are the primary factors influencing ecosystems in highly arid regions, with most species displaying synergistic and positively correlated evolutionary relationships. These findings offer significant insights into the dynamics of zooplankton communities in arid reservoir environments and provide a framework for understanding ecological patterns and community organization under drought conditions.

## 1. Introduction

Northwest China is located in an arid region and is one of the most arid areas in the world. The Tarim River, which is located in the southern part of the Xinjiang Uygur Autonomous Region, flows through the Tarim Basin between the Tianshan and Kunlun Mountains. With a total length of 2179 km, it is the longest inland river in China [1]. The Tarim River originates from the confluence of its main stream and its tributaries, which are mainly fed by glacial meltwater. Its catchment area covers an area of around 1.05 million square kilometers. The Tarim River Basin is located in the heart of the Eurasian continent and is geographically isolated from the ocean and surrounded by mountains. The region experiences significant diurnal temperature fluctuations, with average summer temperatures between 20 °C and 30 °C and average winter temperatures between −10 °C and −20 °C. There is little rainfall, but strong evaporation, so the area is categorized as a warm-temperate arid zone and a typical continental dry climate [2]. Due to the inherent variability and unpredictability of runoff, the main stream and tributaries of the Tarim River face significantly high ecological water supply risks. Although the catchment area has rich groundwater reserves, these are very unevenly distributed and the water has a high degree of mineralization, which makes it difficult to extract and use. Consequently, the construction of reservoirs plays an important role in supporting sustainable development and securing the livelihoods of people in the arid northwest [3].

The Tarim River Basin is a representative freshwater ecosystem in arid Northwest China. This unique geographical location creates a distinctive hydrological and ecological system characterized by extreme environmental conditions [4]. The Tarim River is characterized by sparse vegetation, minimal rainfall and a fragile ecological environment, which contributes to the formation of specialized and fragile ecosystems [5,6]. As a key region of the Belt and Road Initiative, the Tarim River Basin has significant interactions and interdependencies with Northwest China, including in the areas of climate, geomorphology, ecosystems and economic development [7,8]. The water supply function of reservoir groups is a key strategy for alleviating tensions between water supply and demand in river basins. Regulated operation maintains ecological stability in headwater regions without additional water supply, while reducing risks to agricultural water supply along the main stream [9]. Reservoirs serve as critical resource reserves and regulating facilities and play a central role in this region. As important surface water carriers, they enable better support of economic development, improvement of local ecological conditions, and maintenance of the security and stability of the regional ecosystem through the efficient distribution of water resources. The SY, SL, DL and XJZ represent important large-scale freshwater resources in the Tarim River Basin [10].

Zooplankton is widespread in various aquatic ecosystems and is an important component in maintaining the structure and functionality of water ecosystems. As sensitive bioindicators, they react quickly to environmental changes. Environmental factors significantly alter interspecific relationships within zooplankton communities and are an important determinant of zooplankton assemblage dynamics [11]. Zooplankton communities exhibit interspecific interactions characterized by both antagonistic and mutualistic relationships and show complex dynamics between communities [12]. Functional groups refer to assemblages of species that share similar structural characteristics or ecological functions and represent a classification system based on shared characteristics or behaviors within an ecosystem. The study of functional groups offers a more direct approach to clarify how environmental factors influence ecological processes in biological communities. Consequently, the delineation of zooplankton functional groups facilitates the systematic interpretation of relationships between zooplankton communities and ecosystem functioning, thus providing a conceptual framework for understanding the assembly mechanisms of zooplankton communities [13,14]. Several approaches are used to classify plankton communities according to their biological and nutritional characteristics [15,16]. In this study, zooplankton are categorized into functional groups based on life-history characteristics such as body size, diet, mode of reproduction, life cycle pattern, and ability to escape [17]. The structure of the zooplankton community and the mechanisms of its assembly in the arid zones of the high latitudes are still unclear. Current ecological theories suggest that community composition is determined by a combination of deterministic and stochastic factors. The deterministic mechanisms include both biotic interactions (e.g., competition, predation) and abiotic environmental filtering [18,19]. In contrast, stochastic processes indicate random events, such as dispersal limitation and hydrodynamic transport, which significantly influence community composition [20,21]. Consequently, disentangling and quantifying the balance between deterministic and stochastic forces is a crucial approach to deciphering community formation.

In the context of large freshwater bodies in arid Northwest China, these mechanisms and their responses to environmental variability remain poorly understood. Elucidating the rules of plankton assemblage and their spatio-temporal variations in the Tarim River Basin is an important research area to decipher the ecological patterns in the drought-prone aquatic ecosystems of Northwest China. We hypothesized that the structuring and evolutionary dynamics of zooplankton communities in high-latitude arid aquatic systems are mainly determined by three main factors: (1) spatial heterogeneity, (2) seasonal fluctuations, and (3) environmental filtering. The present study aimed to address three fundamental research questions: (a) the specific structural characteristics of plankton communities in reservoir ecosystems under arid conditions; (b) seasonal shifts in the organization of functional groups of plankton communities between hydrological extremes (drought vs. flood periods); (c) the mechanistic driving forces underlying the variability of plankton communities in water-limited reservoir systems. This study aims to explore the assembly mechanisms and key drivers of zooplankton communities in arid regions to provide new insights into the following: (1) understanding ecological patterns and community organization in large water bodies, and (2) developing assessments of aquatic ecosystem health. The results will provide a scientific basis for the systematic monitoring of zooplankton biodiversity and the conservation of fisheries resources in large water bodies. This is the first study to focus on the community classification and zooplankton functional groups in the reservoirs of the Tarim River Oasis in the northwest arid region. It will provide further data on the evolution and functional patterns of zooplankton in the arid regions of Northwest China and even Central Asia.

## 2. Materials and Methods

### 2.1. Study Area

**Figure 1:**
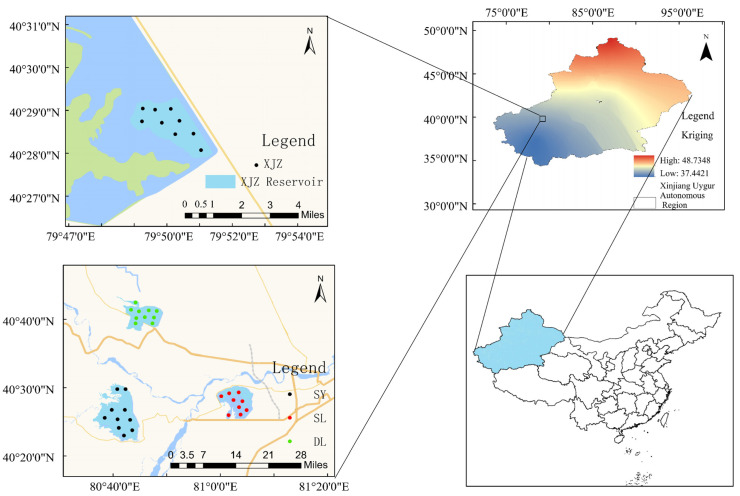
Zooplankton sampling map of Tarim River valley reservoir.

From April 2023 to March 2024, a total of 40 sampling points were set up in four important reservoirs, namely SY, SL, DL and XJZ, in the Tarim River Basin (Figure 1). The flood season in the Tarim River Basin generally occurs during summer. This is mainly due to the significant increase in glacial meltwater following the rise in temperature, coupled with limited rainfall during this period, resulting in abundant river water. Conversely, the low-water period occurs from late autumn to early spring. During this period, the temperature in the runoff-producing area is low; precipitation is sparse and falls mainly as snow that cannot produce direct runoff. Additionally, the seasonal snowpack, glaciers, and permanent snow cover are unable to replenish the river due to low temperatures. [22,23]. Samples were collected in all four seasons: spring (May), summer (July), autumn (October), and winter (November). Spring and summer represent the wet season, while autumn and winter correspond to the dry season.

### 2.2. Sample Collection and Determination Methods

#### 2.2.1. Physicochemical Indices of the Water

The WT (Water Temperature), DO (Dissolved Oxygen), pH, SAL (Salinity), EC (Electrical Conductivity), COND (Conductivity), and TDS (Total Dissolved Solids) data were measured on-site using a water quality detector (the ATI PQ45 multiparameter water quality analyzer from the United States, Beijing Zhongheng Rixin Technology Co., Ltd., Beijing, China). The transparency was measured with a Secchi disk (JCT-8S Secchi disk from Qingdao Juchuang Environmental Protection Group Co., Ltd., Qingdao, China). Indices such as latitude, longitude, and altitude were recorded using a Global Positioning System (GPS, the LS-GPS-639 from Qingdao Lvsheng Biotechnology Co., Ltd., Pingdu, China). The chemical parameters of the water were determined in the laboratory according to the guidelines of *Methods for the Examination of Water and Wastewater (Fourth Edition)* [24].

#### 2.2.2. Zooplankton

The zooplankton was collected using a No. 13 plankton net (with a mesh diameter of 0.112 mm). The sampling bottles were transparent plastic reagent bottles with a wide opening (with a specification of 50 mL). A pipette with a rubber tip (with an inner diameter of 2 mm), a counting chamber (0.1 mL), a cover glass (20 mm × 20 mm), Lugol’s solution, and a 4% formaldehyde solution were used to fix and preserve the zooplankton. The zooplankton were observed under a Leica BME microscope (Leica miorosystems (Shanghai) treadingco., Shanghai, China).

The classification and identification of zooplankton are based on Fauna Sinica—Arthropoda: Crustacea: Freshwater Copepoda; Fauna Sinica—Arthropoda: Crustacea: Freshwater Cladocera; and the *Monograph of the Chinese Freshwater Rotifers* [25,26,27].

The zooplankton was counted using the row-grid method in a zooplankton counting chamber. A 30 mL concentrated water sample was shaken thoroughly and 0.1 mL of the sample was taken with a 0.1 mL pipette and placed in a 0.1 mL counting chamber. A cover glass (22 mm × 22 mm) was placed on it. A total of 30 small grids from the 2nd, 5th, and 8th rows of the counting chamber (the entire slide has 10 rows with a total of 100 grids) were counted by species under a high-power microscope. The counting formula for zooplankton in 1 L of water sample is as follows [28,29,30]:

$$N=\frac{V\cdot P}{W\cdot C}$$

In the formula, the letters are expressed as follows:

*P*: the number of zooplankton observed under the microscope (the average of two slides);

*C*: the volume of the counting chamber (mL);

*V*: the volume of the 1 L water sample after precipitation and concentration (mL);

*W*: the volume of the sampled water (L).

### 2.3. Division of Zooplankton Functional Groups

Zooplankton in freshwater ecosystems are categorized into nine functional groups according to body size, feeding habits, reproductive modes, life cycles, and escape routes: rotifer filter feeders (RF), rotifer predators (RC), rotifer grazers (RP), small zooplankton filter feeders (SCF), small zooplankton predators (SCC), medium-sized zooplankton filter feeders (MCF), medium-sized zooplankton predators (MCC), large zooplankton filter feeders (LCF), and large zooplankton predators (LCC) (Table 1) [11]. In this study, the functional community consisting of 29 dominant species (Y > 0.02) was selected as the object (Table 2), which more intuitively reflects the differences in functional communities under different temporal and spatial changes. The dominant species were categorized into five zooplankton functional groups: rotifer filter feeders (RF), rotifer predators (RC), small zooplankton predators (SCC), medium-sized zooplankton filter feeders (MCF), and medium-sized zooplankton predators (MCC).

### 2.4. Data Processing and Analysis

#### 2.4.1. Calculation of Dominant Species

The calculation formula for the dominant species of zooplankton is as follows [31]:

$$Y=(\frac{n_{i}}{N})f_{i})$$

In the formula, the variables are as follows:

*n_i_*: the proportion of individuals belonging to the *i*-th species relative to the overall number of individuals;

*N*: the total number;

*f_i_*: The frequency of occurrence of the *i*-th species. When *Y* > 0.02, this species is regarded as a dominant species.

#### 2.4.2. Biodiversity

Diversity index of zooplankton (Shannon–Wiener, *H*′).

Shannon–Wiener diversity index (*H*′) calculation formula:

$$H^{\prime }=-\sum p_{i}\times {log}_{2}p_{i}$$

Richness index of zooplankton (Margalef, *d*).

Margalef richness index (*d*) calculation formula:

$$d=(S-1)/{log}_{2}N$$

Evenness index of zooplankton (Pielou, *J*′).

Pielou evenness index (*J*′) calculation formula:

$$J^{\prime }=H^{\prime }/{log}_{2}S$$

Zooplankton diversity index (Simpson, *D*).

Simpson diversity index (*D*) calculation formula:

$$D=1-\sum {Pi}^{2}$$

*S*: the number of species in the community;

*N*: the total number of individuals observed in the quadrat;

*Pi*: the proportion of the number of individuals of the *i*-th species at this station out of the total number of individuals.

In the above analysis, PRIMER 5.2.8 was used to calculate the Margalef species richness index (*d*), the Shannon–Wiener diversity index (*H*′), the Simpson dominance index (*D*), and the Pielou evenness index (*J*′) to measure and analyze the biodiversity of the zooplankton community [32].

#### 2.4.3. Canonical Correspondence Analysis (CCA)

A DCA (Detrended Correspondence Analysis) was carried out using the Canoco 5 software for Windows 4.5. The result showed that the SD (standard deviation) value was greater than 3. Therefore, CCA (canonical correspondence analysis) was performed with the data of species and relevant environmental factors to analyze the effects of several environmental factors on zooplankton [33].

#### 2.4.4. Bray–Curtis Similarity Analysis

The Bray–Curtis similarity coefficient was calculated based on the biomass of the zooplankton functional groups. SPSS Statistics 27 software was used, and MDS (Multidimensional Scaling) was applied to analyze the difference between the zooplankton functional groups in the different seasons and at the different sampling sites [34].

#### 2.4.5. Pearson Correlation Analysis

After applying a log_10(x+1)_ transformation to the functional group of zooplankton data to improve normality, a Pearson correlation analysis using the average clustering method and the Euclidean distance algorithm was performed to investigate the interaction between the different zooplankton functional groups and their influence on environmental factors [35].

#### 2.4.6. Prediction of New Random Forest Model

Using R version 3.1, the new package Random Forest (rfPermute) was used for the corresponding analysis. The R value was calculated with the package tidyverse, and the plot exploration was performed with the R package ggplot 2. Error was calculated using out-of-bag (OOB) data, which determined the relative importance of each functional group in the analysis, which was then ranked [36].

#### 2.4.7. Collinear Network of Zooplankton Functional Groups

To explore the functional relationships between the zooplankton communities from different angles, a correlation analysis of the collinear network was performed using Kendall’s method, with a correlation threshold of 0.6 and a *p*-value threshold of 0.001. The analysis results of the microbial co-occurrence network were generated using the R 4.3.1 software package “WGCNA”.

## 3. Results

### 3.1. Composition of Functional Groups of Zooplankton

**Figure 2:**
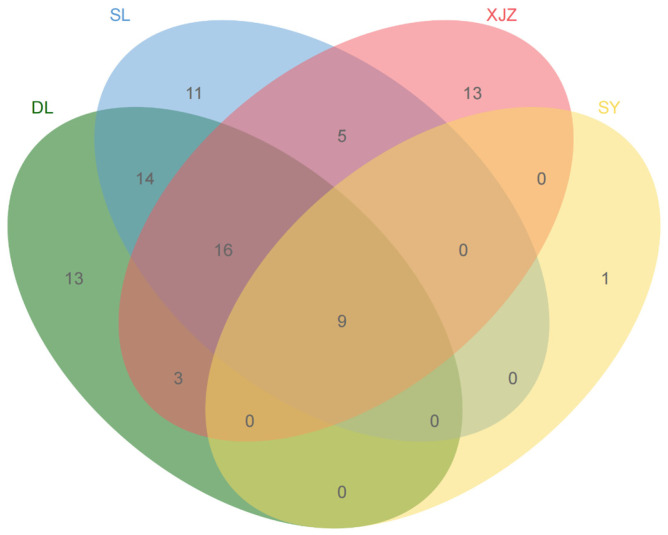
Venn diagram of plankton species composition in four affiliated reservoirs of the Tarim River. Note: The numbers indicate the number of species endemic to the region in a single color, and the numbers indicate the number of species common to the corresponding region in the cross color.

In this study, 85 species of zooplankton were identified at 40 sampling sites during the dry and wet seasons (Figure 2), including 56 species of rotifers accounting for 65.9%, 17 species of Cladocera accounting for 20%, and 12 species of copepods accounting for 12.1%. There are considerable differences in species composition between the four reservoir areas, with a total of nine common species. SL has 11 unique species; XJZ and DL each have 13 unique species; and there is only 1 species unique to SY. Table 3 shows the interaction relationships between the zooplankton functional groups. In order to reduce the complexity of the interactions and to more intuitively reflect the differences in the functional communities with temporal and spatial changes, only dominant species with a degree of dominance of more than 0.02 were retained as functional group taxa, and a total of five functional groups were subdivided (Table 2).

**Figure 3:**
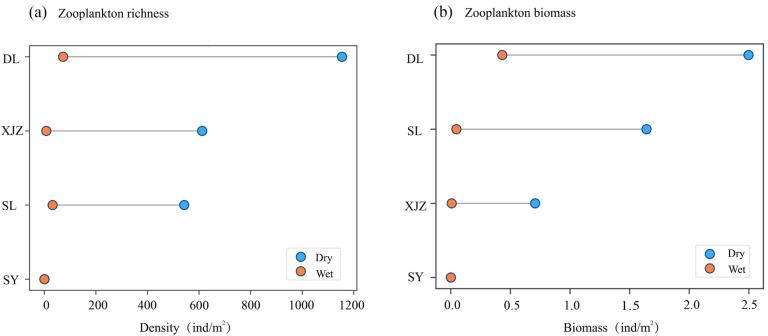
Spatial–temporal variation diagram of zooplankton richness and biomass in four affiliated reservoirs of the Tarim River. Note: (**a**) shows the density of zooplankton; (**b**) shows the biomass of zooplankton.

The temporal and spatial changes in zooplankton abundance are shown in Figure 3a. From a temporal perspective, the range of seasonal changes in the species abundance of zooplankton in SY is relatively small. In the other reservoirs, the seasonal variation shows that the abundance range during the wet season is much higher than that during the dry season. Among them, the range of changes in species abundance between the dry and wet seasons is the widest in DL. Analyzing from the perspective of spatial distribution, the abundance range in DL is the widest. The abundance ranges in SL and XJZ are relatively similar. As the water inlet, SY has its water replenished by the Tarim River all year round due to its unique geographical location, and its abundance range is significantly lower than that of the other three reservoirs.

The temporal and spatial changes in the biomass of zooplankton are shown in Figure 3b. From a temporal perspective, the range of biomass changes in SY between the dry and wet seasons is relatively small. For the other three reservoirs, the range of biomass changes shows that the values in the wet season are much higher than those in the dry season, and the overall trend is consistent with that of the abundance. The density of SL is lower than that of XJZ, but its biomass is higher, suggesting that the organisms in SL have larger volumes and stronger feeding abilities. In terms of horizontal spatial distribution, the biomass range in DL is much wider and higher than that in the other three reservoirs, while the biomass range in SY is significantly narrower and lower than that in the other three reservoirs.

**Figure 4:**
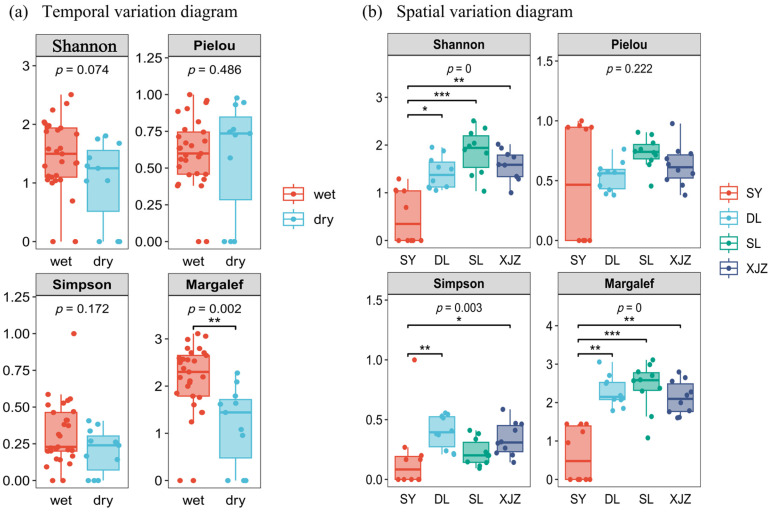
Boxplot of alpha diversity index for zooplankton. Note: (**a**) represents the α diversity in zooplankton time changes; (**b**) represents the α diversity in zooplankton spatial changes. * significant at the 0.05 level; ** very significant at the 0.01 level; *** very significant at the 0.001 level. Shannon Diversity Index (*H*^′^); Pielou Uniformity Index (*J*); Simpson Advantage Index (*D*); Margalef Richness Index (*d*).

The temporal and spatial changes in the diversity of zooplankton in the connected reservoirs of the Tarim River are shown in Figure 4. From the perspective of the temporal scale, diversity indices such as Shannon, Simpson, and Pielou show relatively small differences. Only the Margalef richness index (*p* < 0.01) shows a large seasonal variation, as it is much higher in the wet season than in the dry season. When analyzed from a spatial perspective based on the Shannon diversity index (*p* < 0.01), the value in SL is higher than in the other three regions. This is followed by DL and XJZ, and the value in the SY region is much lower than the three regions. These differences indicate considerable spatial variations in zooplankton diversity in the reservoirs. The Pielou evenness index shows only slight differences. In contrast, the Simpson dominance index (*p* < 0.05) is highest in DL, followed by XJZ and SL, and lowest in the SY region. Similarly, the Margalef richness index (*p* < 0.01) is also slightly higher in SL than in the DL and XJZ regions, with SY having significantly lower values than the other three regions.

An analysis of the classification of the diversity index of the zooplankton community and the abundance index shows these significant differences between the four connected reservoirs of the Tarim River. The specific values range between 1.67 and 3.49 (diversity index) and between 1.67 and 14.95 (richness index). In the XJZ region, the diversity index is at a “good” level during the wet season and the richness index is at a “high” level; during the dry season, both indices fall to a “medium” level. In the SY region, the diversity index is at a “medium” level during the wet season and the richness index is at a “poor” level. During the dry season, the diversity index is at a “low” level and the richness index is also at a “poor” level. The SL region has a “good” diversity index and a “high” richness index during the wet season. During the dry season, the diversity index drops to a “low” level and the richness index to a “medium” level. The DL region has a “good” diversity index and the richness index is at a “high” level in the wet season, whereas both indices fall to a “medium” level in the dry season.

### 3.2. The Spatiotemporal Heterogeneity and Similarity Coefficients of Functional Groups of Zooplankton

**Figure 5:**
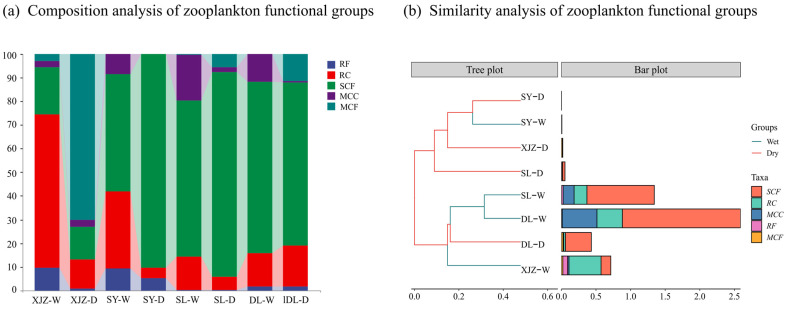
Composition and similarity analysis of zooplankton functional groups. Note: (**a**) percentage of species composition in zooplankton functional groups; (**b**) species composition and similarity analysis representing functional groups of zooplankton.

The 29 dominant species with a zooplankton dominance of more than 0.02 were categorized into five functional groups (Table 2). The spatial and temporal distribution of the zooplankton functional groups is shown in Figure 5. The seasonal differences in the average biomass of the zooplankton functional groups in DL and SL are relatively small, and both are mainly dominated by the functional group SCF. In contrast, the seasonal differences in XJZ and SY are relatively large. In XJZ, the predominant functional group in the wet season is RC, while in the dry season, it changes to MCF. In SY, RC and SCF dominate in the wet season with 49.60% and 32.64%, respectively, while in the dry season, SCF is the absolutely dominant group with 90.28% (Figure 5a). The Bray–Curtis similarity coefficient was used to analyze the similarity in the composition of zooplankton functional groups in the different reservoirs (Figure 5b), and the results are broadly consistent with the trends in temporal and spatial variables of zooplankton abundance and biomass. There are extremely significant differences in the biomass of the zooplankton functional groups between the four reservoirs, with DL having a significantly higher biomass than the others. The greatest similarity in biomass exists between the zooplankton functional groups of SY and DL, although the biomass in SY is significantly lower than in the other reservoirs. The highest similarity index for the composition of zooplankton functional groups was between the flood seasons in SL and DL. The similarity index between the flood season and the dry season in SY ranked second, indicating that the seasonal changes in the composition of zooplankton functional groups in SY are not obvious. Overall, the functional group SCF is the dominant group in all four regions.

**Figure 6:**
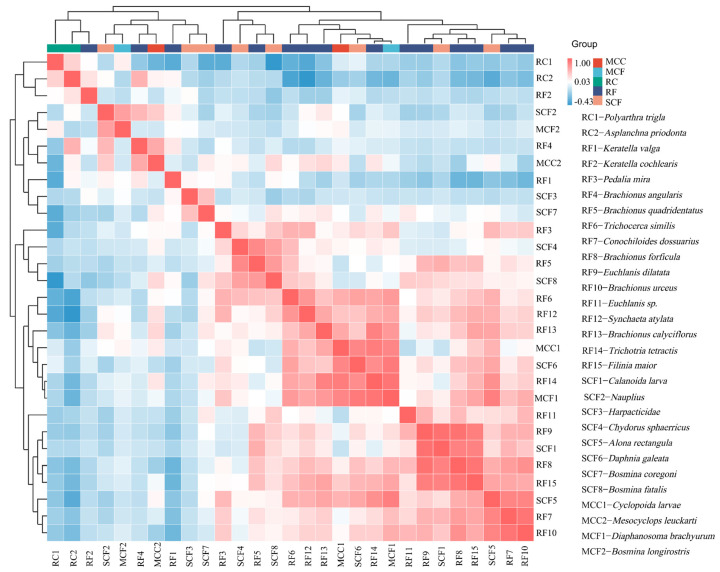
Interaction diagram of dominant zooplankton species in four affiliated reservoirs of the Tarim River.

Figure 6 shows the positive and negative correlations between 29 species of zooplankton in five functional groups. Most species within the same functional groups show negative correlations. In contrast, the relationships between zooplankton of different functional groups show a positive correlation, and the most obvious one is between RF and SCF.

### 3.3. Study on the Analysis of Physical and Chemical Indexes of Water from a Multidimensional Perspective

**Figure 7:**
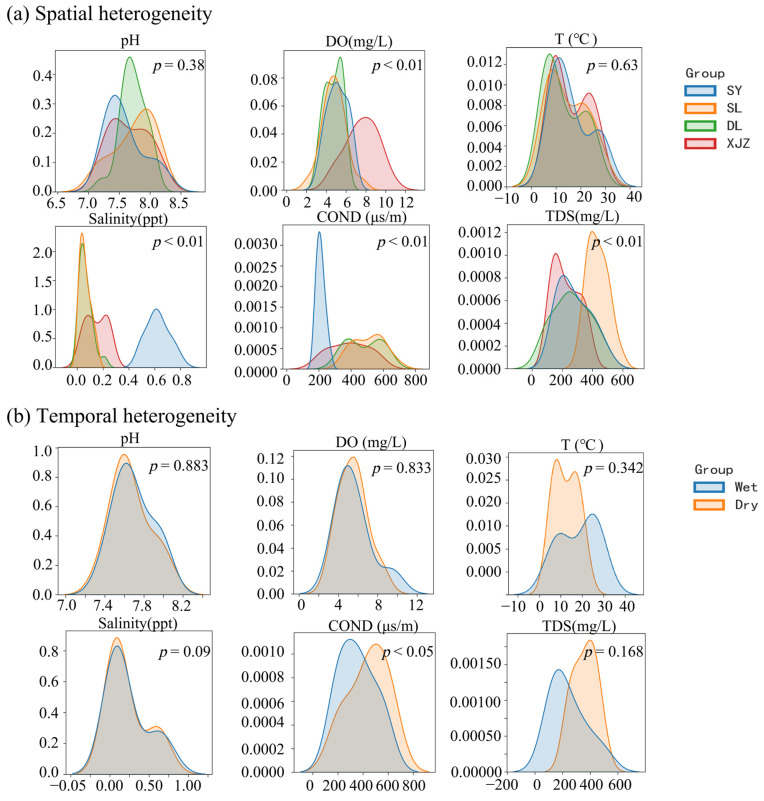
Analysis of the spatial–temporal differences in physical and chemical indexes of water bodies in four affiliated reservoirs of the Tarim River. Note: (**a**) shows the spatial changes of environmental indicators; (**b**) shows the time change of environmental indicators.

The spatial variation in the water environment factors is shown in Figure 7a. The results of the ANOVA show that, with the exception of temperature and pH, all other environmental factors exhibit spatial variation. DO is significantly higher in XJZ compared to the other three reservoirs; SY has significantly higher SALIN than the other three reservoirs, indicating a trend towards saline–alkali water, and also has the lowest COND; SL has the highest TDS. The seasonal variation in water environmental factors is shown in Figure 7b. pH, DO, and SALIN are highly overlapping in the water environment and show a relatively stable seasonal pattern. COND and TDS are significantly higher in the dry season than in the wet season, while temperature is significantly lower in the dry season. Collectively, these findings indicate that the environmental factors show seasonal variation.

**Figure 8:**
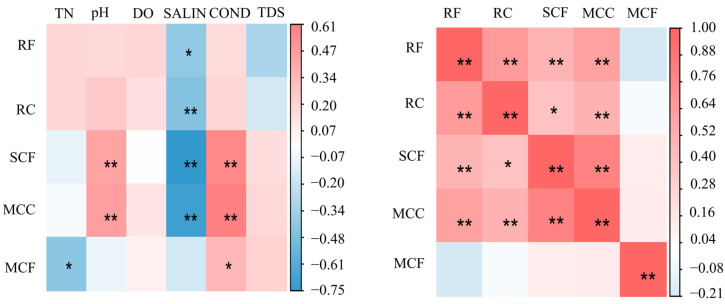
Correlation analysis among zooplankton functional groups and between zooplankton functional groups and water environmental factors in four affiliated reservoirs of the Tarim River. Note: (**a**) shows the correlation analysis between functional groups and environmental factors of zooplankton; (**b**) shows the correlation analysis within the functional population of zooplankton * significant at the 0.05 level; ** very significant at the 0.01 level.

Pearson correlation analysis showed that the biomass of zooplankton functional groups was not correlated with most water environmental factors (Figure 8a) (*p* > 0.05); it was only positively correlated with pH and COND (*p* < 0.05) and negatively correlated with SALIN (*p* < 0.05). SCF showed a highly significant negative correlation with SALIN (*p* < 0.01). Both MCC and SCF showed a significant positive correlation with pH (*p* < 0.01); MCF was positively correlated with COND (*p* < 0.05) and negatively correlated with TN (*p* < 0.05); and RF, apart from its relationship with SALIN, was not significantly correlated with other water environmental factors. Compared with water environmental factors, the significance was more obvious among zooplankton functional groups (Figure 8b). Except for MCF, the functional groups RF, RC, SCF, and MCC were significantly or extremely significantly correlated with each other.

**Figure 9:**
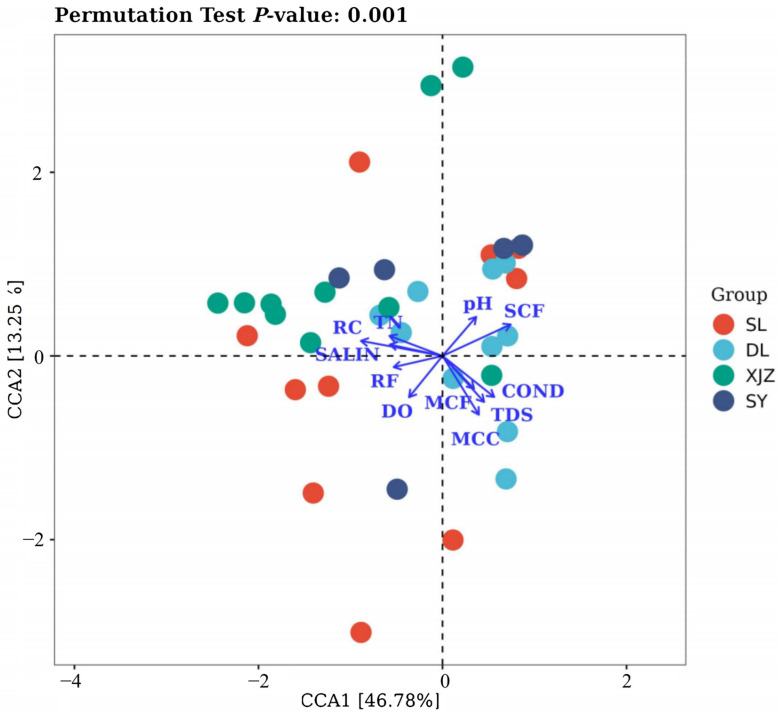
CCA (canonical correspondence analysis) of zooplankton functional groups and environmental factors in four affiliated reservoirs of the Tarim River.

The effects of environmental factors on zooplankton were investigated (Figure 9). The explanatory rate of CCA1 was 46.78%, the explanatory rate of CCA2 was 13.25%, and the cumulative explanatory rate of CCA1 and CCA2 was 60.03%. The biomass of zooplankton functional groups was mainly affected by pH, COND, RC, SCF, SALIN, and MCC, and other factors, specifically RC and MCC, were positively correlated with pH, RF, SCF, and MCC. Only RF showed a positive correlation with the functional groups SCF, MCC, and RC, but it showed no significant correlation with water environmental factors. SCF displayed a negative correlation with SALIN and a positive correlation with all other functional groups except MCF. MCF was only positively correlated with COND. Overall, most environmental factors have no obvious correlation with the biomass of zooplankton functional groups, while the relationships among zooplankton functional groups are more significant.

### 3.4. Predictive Modeling and Quantitative Evaluation of Functional Group Niches

**Figure 10:**
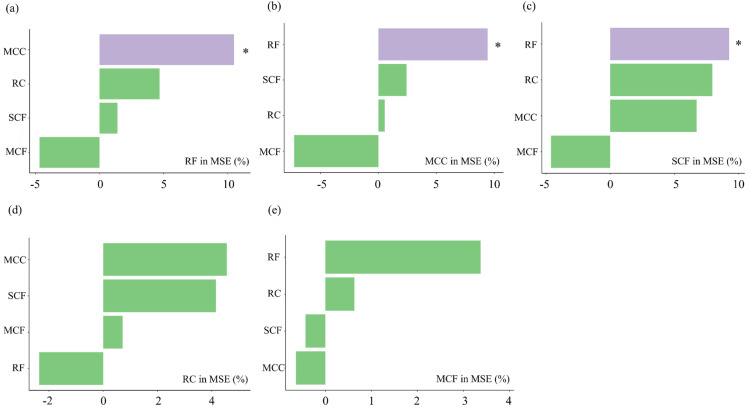
Prediction of interaction strength among plankton functional groups in four affiliated reservoirs of the Tarim River based on the random forest model. Note: (**a**) the importance of the MCC, RC, SCF, and MCF functional groups to the RF functional group; (**b**) the importance of the RF, RC, SCF, and MCF functional groups to the MCC functional group; (**c**) the importance of the MCC, RC, RF, and MCF functional groups to the SCF functional group; (**d**) the importance of the MCC, RF, SCF, and MCF functional groups to the RC functional group; (**e**) the importance of the MCC, RC, SCF, and RF functional groups to the MCF functional group. * and purple significant at the 0.05 level.

Random forest analysis (Figure 10) revealed key interactions between zooplankton functional groups. The most influential factor for RF is MCC, indicating a strong positive correlation. MCF had a negative correlation with RF, MCC, and SCF. The RF functional group was the most important factor influencing the SCF functional group, and they were positively correlated. Among all interactions, the functional group RC demonstrated the highest positive correlation with MCC and a negative correlation with RF. Additionally, the MCF functional group had the highest positive correlation with RF and a negative correlation with both MCC and SCF.

**Figure 11:**
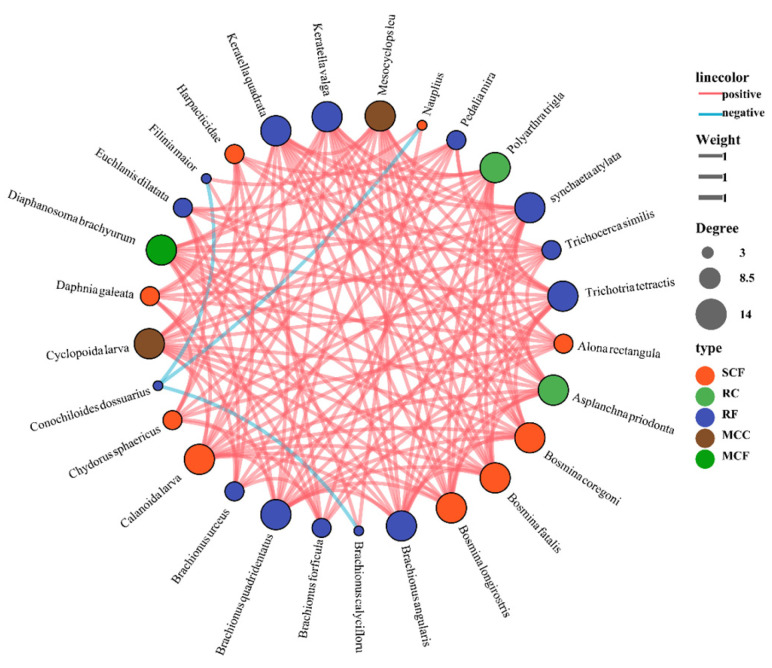
Collinearity network diagram of plankton functional communities in four affiliated reservoirs of the Tarim River. Note: the correlation threshold is 0.6, and the *p*-value threshold is 0.001.

The network diagram presented in Figure 11 illustrates the co-occurrence patterns among zooplankton, revealing the structure, functions, and ecological characteristics of the zooplankton community. The network diagram of zooplankton is dominated by positive edges, indicating that facilitative interactions are the primary survival strategies within the community. The structure is primarily driven by the SCF and RF functional groups, which play central roles in maintaining network connectivity. The probability of mutual connection among the neighbors of nodes reflects the clustering degree of the network. The ratio of the actual number of edges in the network to the total number of possible edges further highlights the close degree of the interactions among zooplankton.

## 4. Discussion

### 4.1. Analysis of Composition and Diversity of Zooplankton Communities in Arid Areas

Zooplankton are highly sensitive to environmental changes and are therefore considered good indicators of an ecosystem. Changes in the physical, chemical, and biological parameters of aquatic systems lead to variations in the relative composition and abundance of plankton [37,38]. Scholars such as Wassim Guermazi have shown that freshwater zooplankton are mainly composed of rotifers and small crustaceans (Cladocera and Copepoda) [39]. In terms of biodiversity in the affiliated reservoirs of the Tarim River Basin, the number of rotifer species is much higher than that of cladocerans and copepods, which is consistent with survey results from other scholars on the DL [40]. Changes in the relative and absolute biomass of zooplankton communities can affect productivity at higher trophic levels by altering secondary production rates and energy transfer efficiency [41]. In this study, cladocerans are the zooplankton with the most dominant quantity, with *Bosmina coregoni* being the absolutely dominant species, almost entirely representing the SCF functional group. Cladocerans have a relatively large body size and play an important role in aquatic ecosystems. On the one hand, they can directly affect the abundance of phytoplankton in the water. On the other hand, they can serve as natural food sources for fish and other aquatic organisms [42,43]. Branchia and cladocera feed on bacteria, algae, rotifers, and protozoa. The rotifer species are dominated by needle cluster *polybranch rotifers* [11]. As a food source for medium-sized and large zooplankton, rotifers transfer energy and organic matter to higher trophic levels, thus completing the energy cycle and material transformation within the zooplankton community, in turn supporting the favorable construction of the zooplankton community [44,45]. Diversity indices play an important role in evaluating the stability of zooplankton communities and the quality of the ecological environment [46]. In their studies, Chainho et al. divided the *H*′ and *d* of zooplankton communities into five distinct grades (Table 3), including high, good, medium, low, and poor [47]. After comparing with the water pollution degree (Table 4), it was revealed that the zooplankton’s *H′* values consistently indicated lower pollution levels compared to *d* values in each region. Overall, the pollution types of the Tarim River are quite different. Except the SY area during the flood season, all areas are classified as the clean to oligopollutant type, while the SY area is in the β medium pollution type; the dry season is in the β medium pollution type to α medium pollution–heavy pollution type. There are significant differences in diversity between the wet season and the dry season across the four reservoirs. The reason for this result may be due to intensified photosynthesis during summer, leading to the significant growth of phytoplankton. With an abundance of food sources, zooplankton populations increase significantly, resulting in higher diversity and richness indices in the wet season compared to the dry season. This research finding is consistent with the research results of studies conducted in other reservoirs [48,49].

**Table 3:** Classification of diversity index and abundance index of zooplankton community [47].

Classifications	Diversity Index (*H*′)	Richness Index (*d*)
High	>4.0	>4.0
Good	3.0~4.0	>4.0
Moderate	2.0~3.0	2.5~4.0
Poor	1.0~2.0	<2.5
Bad	0.0~1.0	<2.5

### 4.2. Changes in Functional Community Characteristics of Zooplankton

The densities and biomasses of zooplankton vary among different habitats, and the compositions of zooplankton are also different [51,52,53]. The affiliated reservoirs in the Tarim River Basin have a unique geographical location and are rarely disturbed by human activities. Their hydrological environment is closely related to that of the Tarim River. Frequent changes in water levels within the reservoirs result in significant fluctuations in water depth. Moreover, these reservoirs are mainly connected to the SY, leading to a significantly lower quantity of zooplankton in the SY compared to other areas. This finding is consistent with the results of Gutierrez et al., who observed zooplankton in the humid areas downstream from the Paraná River Delta during an extreme drought event. During periods of low water levels, zooplankton showed higher taxonomic diversity along with significant changes in biomass [54].

The results of the Bray–Curtis analysis showed significant differences in the functional groups of zooplankton in the affiliated reservoirs in the Tarim River Basin, varying by region and season, and the composition of the zooplankton community throughout the basin was not uniform, showing temporal and spatial differences in species richness and biomass [55]. The survey shows that during the wet season, rising temperature and increased light intensity accelerate the growth rate of zooplankton. Dormant eggs float to the water surface and hatch, leading to a rapid increase in species number, which promotes the improvement of diversity [56,57,58]. The SCF functional group of zooplankton almost completely dominates across different regions and times. Moreover, during the wet season, the relative biomasses of the filter-feeding functional groups RF and SCF further increase. This may be due to the higher water temperature during this period, which promotes the growth and reproduction of algae, providing better food availability. Therefore, these groups are dominant during the wet season. The SCF group feeds on bacteria, algae, organic matter, and protozoa, and holds a dominant position in the aquatic ecosystem. Therefore, it is speculated that the SCF functional group of zooplankton plays an important role in regulating aquatic ecosystems at high latitudes [59,60].

### 4.3. Interaction of Zooplankton Functional Groups and Their Relationship with Water Environmental Factors

In recent years, research on zooplankton inter specific interactions has advanced, but the study of zooplankton in high-latitude arid areas remains limited [61,62]. Species with a broad ecological niche exhibit strong environmental adaptability. However, as population density increases, the competition between species also intensifies accordingly. Competition and predation behavior among zooplankton species are key drivers of population dynamics [63]. Functional groups occupying different ecological niches may engage in predator–prey relationships, creating bidirectional influences that shape community structure [64]. These dynamics are further modulated by upward effects such as temperature, nutrient salts and phytoplankton availability, downward effects of fish predation, interspecific competition, etc. Collectively, these factors govern zooplankton growth and the spatial distribution of zooplankton functional groups [65].

The functional groups RF, SCF, and MCF primarily feed on bacteria and algae. Fluctuations in these food sources will directly impact filter-feeding zooplankton populations and have an indirect effect on predatory zooplankton through trophic cascades. As key primary producers, phytoplankton can effectively regulate the balance of aquatic ecosystems. Temperature is an important environmental factor affecting the growth of phytoplankton [66,67]. Zooplankton functional groups have a strong responsiveness to changes in phytoplankton [68]. Evans L. E. et al. conducted 57-year monitoring study of zooplankton in the Atlantic Ocean, revealing a negative correlation between copepod community body size and surface temperature, consistent with Bergman’s law [69]. However, through CCA, this study found that temperature is not the main environmental factor affecting the composition of the zooplankton community. Instead, water chemistry parameters, particularly pH (the most influential driving factor), COND, and SALIN, have a stronger effect on zooplankton functional groups. The pH value is a key variable of zooplankton functional diversity, demonstrating strong correlations with all measured indicators; even minor pH fluctuations significantly affect the functional diversity index. In arid regions, where soil usually exhibits alkaline or strongly alkaline properties, osmosis and wind–sand erosion lead to a significant increase in the alkalinity of surrounding water bodies [70]. Branching and radulids have a competitive advantage over smaller species such as rotifers through their higher filtration rates and the ability to collect resources [71]. Extreme pH values (either high or low) will affect the ionization of organic matter in aquatic systems, thereby indirectly affecting the growth of algae. If the pH value of water is too high, the number of cyanobacterial species will increase [72,73]. This trophic dynamic favors energy flow toward larger predators, particularly enhancing the development and population density of medium-to-large SCF and MCC communities [74]. In addition, aquatic organisms must adapt to water level fluctuations that further modulate pH variability [75]. In this study, the pH level was higher in the wet season compared to the dry season, which may be related to the increase in free CO_2_ absorption by the water environment due to the enhanced photosynthetic activity, resulting in the occurrence of a high pH value in the water body [76,77]. The biomass of zooplankton across the four reservoirs showed no significant seasonal variation with pH changes, but the higher standard deviation of zooplankton biomass during dry seasons indicates that even a minor fluctuation in pH would produce significant differences in the biomass of the zooplankton community in the dry season [78]. These findings align with research by Ruizhi An in the alpine wetlands of the Qinghai–Tibet Plateau (similarly frigid and arid ecosystems), where ecosystems demonstrate exceptional sensitivity to climate change, with pH and SALIN being the dominant controlling factors [79]. This finding further demonstrates that ecosystems in high-latitude arid regions are extremely sensitive to climate change, largely due to the sensitivity of zooplankton to factors such as SALIN and pH. Pearson correlation analysis shows that interspecific competition is also one of the main factors affecting zooplankton functional groups [80]. Through the further classification of zooplankton functional groups, there are significant correlations among the functional groups RC, RF, SCF, and MCC, while MCF showed no apparent relationships with other groups. Except for XJZ, SCF is the dominant group in the other three reservoirs. Some studies have pointed out that there is a negative correlation between the body size of zooplankton and food resources, and the predation of fish also leads to a reduction in the body size of zooplankton [81]. XJZ is a medium-sized plain reservoir, significantly smaller in area and higher in fish abundance than the other three reservoirs [49].

This distinction in XJZ is different from the other three reservoirs. This is consistent with the study by De Meester L., stating that fish population composition will directly affect the community composition of zooplankton [82]. Further research on inter-species relationships was conducted by performing clustering and Pearson correlation analysis on dominant species with a dominance greater than 0.02. The higher the similarity between species, the higher the negative correlation they exhibit, as their overlapping ecological niches (including similar life histories and feeding habits) will intensify the competition and predation pressure. The low similar species occupy different ecological niches primarily demonstrated positive correlation through trophic or mutualistic relationships [83]. The growth of zooplankton is jointly influenced by factors such as the flow velocity of water bodies; the nutritional status of water quality, temperature, pH value, and phytoplankton; and the pressure from predators [84]. The factors influencing zooplankton functional groups in the arid regions of Northwest China are mainly the interactions among the zooplankton functional groups and water environmental factors, and the influence among the functional groups is more significant.

### 4.4. Quantitative Evaluation of Functional Group Ecological Niche Contribution Model

In this study, the random forest model was used to predict the importance index of interactions among zooplankton functional groups. For large and medium-sized zooplankton such as MCC, SCF, and MCF, the functional group RF is the most important positively correlated factor. For RF and RC, MCC is the most significant positive influencing factor. However, the functional group MCF has an inhibitory effect on RF, MCC, and SCF, showing a negative correlation. According to the model prediction, an increase in the RF functional group leads to rapid growth in large and medium-sized zooplankton such as MCC, SCF, and MCF. MCC and MCF feed on bacteria, fungi, and small rotifers. When RF increases, there is an abundant supply of food. This reduces competition among these groups, allowing their populations to increase simultaneously. However, once MCF reaches a certain threshold, it competes with SCF and MCC (which also rely on RF as a food source), ultimately suppressing the numbers of SCF and MCC. Additionally, RF may be preyed upon by MCF, causing its population to decline correspondingly as MCF increases. To analyze the relationships among zooplankton functional communities from different perspectives, the Kendall method was adopted for calculating the collinear network correlation, with a correlation threshold of 0.6 and a *p*-value threshold of 0.001. The zooplankton network diagram is dominated by positive edges, indicating that facilitative interactions (mutual promotion) are the primary survival strategy within the community. The network’s connectance, defined as the ratio of the actual edges to the number of all possible edges, reflects the closeness of zooplankton interactions [85]. From the perspective of survival strategies, the relationships among zooplankton are quite complex. Most of these are facilitative, enabling mutualistic support to cope with external environmental pressures. Among species with a high degree of overlap in their ecological niches, competition for nutritional resources exhibits strong asymmetric characteristics [86]. Under homogenizing selective pressures, the top–down control by fish, and the trophic cascades of zooplankton, functionally redundant species exhibit alternative competitive responses. Specifically, it is manifested as follows: The biomass fluctuation in a single population can trigger a cascading effect, and resource partitioning reconstruction initiates multi-trophic feedback across dynamic responses at multiple trophic levels [87,88]. This dynamic equilibrium is constrained by the two-way regulation of competitive exclusion among species and co-evolution; ultimately these processes generate a nonlinear community succession pattern [89].

## 5. Conclusions

In this study, a total of 85 species of zooplankton were identified from 40 sampling points during both the dry and wet seasons. The number of rotifer species was much higher than that of cladocerans and copepods, but the cladocerans dominated in both density and biomass. The zooplankton were classified into five functional groups, with the SCF functional group showing clear dominance. The water quality correlation analysis revealed that pH and SALIN are the primary factors influencing the ecosystem in high-altitude arid regions. Species interaction analysis indicated that most species exhibit a synergistic and positively correlated evolutionary relationship.

## Figures and Tables

**Table 1:** Description of zooplankton functional groups in freshwater ecosystems [11].

Functional Group	Size/mm	Feeding Habits
Rotifer filter feeders, RF *	-	They feed on bacteria, algae, and organic matter
Rotifer carnivora, RC *	-	They feed on protozoa, other rotifers, and small crustaceans
Rotifer predators, RP	-	They feed mainly on algae
Small copepods and claocera filter feeders, SCF *	<0.7	They feed on bacteria, algae, organic matter, and protozoa
Small copepods and claocera carnivora, SCC	<0.7	They feed on rotifers, cladocera, dipteran insects (larvae of mosquitoes), and oligochaetes
Middle copepods and claocera filter feeders, MCF *	0.7~1.5	They feed on bacteria, algae, organic matter, and protozoa
Middle copepods and claocera carnivora, MCC *	0.7~1.5	They feed on rotifers, cladocera, dipteran insects (larvae of mosquitoes), and oligochaetes
Large copepods and claocera filter feeders, LCF	>1.5	They feed on bacteria, algae, organic matter, and protozoa
Large copepods and claocera carnivora, LCC	>1.5	They feed on rotifers, cladocera, dipterans (larvae of midges), and oligochaetes

Note: for the functional groups assigned in this study that are marked with *, this indicates that the size was not calculated for the feeding-habit grouping.

**Table 2:** Functional groups of zooplankton in the Tarim River Basin.

Species	Class	Functional Groups	Figure 6 Code
***Polyarthra trigla***	Rotifera	RC	RC1
***Asplanchna priodonta***	Rotifera	RC	RC2
***Keratella valga***	Rotifera	RF	RF1
***Keratella cochlearis***	Rotifera	RF	RF2
***Pedalia mira***	Rotifera	RF	RF3
***Brachionus angularis***	Rotifera	RF	RF4
***Brachionus quadridentatus***	Rotifera	RF	RF5
***Trichocerca similis***	Rotifera	RF	RF6
***Conochiloides dossuarius***	Rotifera	RF	RF7
***Brachionus forficula***	Rotifera	RF	RF8
***Euchlanis dilatata***	Rotifera	RF	RF9
***Brachionus urceus***	Rotifera	RF	RF10
***Euchlanis* sp.**	Rotifera	RF	RF11
***Synchaeta atylata***	Rotifera	RF	RF12
***Brachionus calyciflorus***	Rotifera	RF	RF13
***Trichotria tetractis***	Rotifera	RF	RF14
***Filinia maior***	Rotifera	RF	RF15
***Calanoida larva***	Copepoda	SCF	SCF1
***Nauplius***	Copepoda	SCF	SCF2
***Cyclopoida larvae***	Copepoda	MCC	MCC1
***Harpacticidae***	Copepoda	SCF	SCF3
***Mesocyclops leuckarti***	Copepoda	MCC	MCC2
***Chydorus sphaerricus***	Cladocera	SCF	SCF4
***Alona rectangula***	Cladocera	SCF	SCF5
***Diaphanosoma brachyurum***	Cladocera	MCF	MCF1
***Daphnia galeata***	Cladocera	SCF	SCF6
***Bosmina longirostris***	Cladocera	MCF	MCF2
***Bosmina coregoni***	Cladocera	SCF	SCF7
***Bosmina fatalis***	Cladocera	SCF	SCF8

**Table 4:** Relationship between plankton diversity index, nutrient levels, and water pollution types [50].

Types of Water Pollution	Diversity Index (*H*′)	Richness Index (*d*)
Oligosaprobic	>4.0	>4.0
Lightly polluted	>3.0~4.0	>3~4.0
β-Mesosaprobic	>1.0~3.0	2~3
α-Mesosaprobic to polysaprobic	0~1	0~2

## Data Availability

The data supporting this study’s findings are available from the corresponding authors upon reasonable request.
